# Cognitive Behavioural Therapy for Nightmares for Patients with Persecutory Delusions (Nites): An Assessor-Blind, Pilot Randomized Controlled Trial

**DOI:** 10.1177/0706743719847422

**Published:** 2019-05-26

**Authors:** Bryony Sheaves, Emily A. Holmes, Stephanie Rek, Kathryn M. Taylor, Alecia Nickless, Felicity Waite, Anne Germain, Colin A. Espie, Paul J. Harrison, Russell Foster, Daniel Freeman

**Affiliations:** 1 Department of Psychiatry, Warneford Hospital, University of Oxford, Oxford, UK.; 2 Oxford Health NHS Foundation Trust, Warneford Hospital, Oxford, UK.; 3 Department of Psychology, Uppsala University, Sweden.; 4 Division of Psychology, Department of Clinical Neuroscience, Karolinska Institutet, Solna, Sweden.; 5 Nuffield Department of Primary Care Health Sciences, Primary Care Clinical Trials Unit, University of Oxford, Radcliffe Observatory Quarter, Oxford, UK.; 6 Department of Psychiatry, University of Pittsburgh School of Medicine, University of Pittsburgh, Pittsburgh, PA, USA.; 7 Nuffield Department of Clinical Neurosciences, University of Oxford, Oxford, UK.

**Keywords:** nightmares, psychosis, paranoia, sleep, schizophrenia, mental imagery

## Abstract

**Objective:**

Nightmares are relatively common in patients experiencing psychosis but rarely assessed or treated. Nightmares may maintain persecutory delusions by portraying fears in sensory-rich detail. We tested the potential benefits of imagery-focused cognitive behavioural therapy (CBT) for nightmares on nightmare severity and persecutory delusions.

**Method:**

This assessor-blind parallel-group pilot trial randomized 24 participants with nightmares and persecutory delusions to receive CBT for nightmares delivered over 4 weeks in addition to treatment as usual (TAU) or TAU alone. Assessments were at 0, 4 (end of treatment), and 8 weeks (follow-up). Feasibility outcomes assessed therapy uptake, techniques used, satisfaction, and attrition. The primary efficacy outcome assessed nightmare severity at week 4. Analyses were intention to treat, estimating treatment effect with 95% confidence intervals (CIs).

**Results:**

All participants offered CBT completed therapy (mean [SD], 4.8 [0.6] sessions) with high satisfaction, and 20 (83%) participants completed all assessments. Compared with TAU, CBT led to large improvements in nightmares (adjusted mean difference = −7.0; 95% CI, –12.6 to –1.3; *d* = –1.1) and insomnia (6.3; 95% CI, 2.6 to 10.0; *d* = 1.4) at week 4. Gains were maintained at follow-up. Suicidal ideation was not exacerbated by CBT but remained stable to follow-up, compared with TAU, which reduced at follow-up (6.8; 95% CI, 0.3 to 3.3; *d* = 0.7). CBT led to reductions in paranoia (–20.8; 95% CI, –43.2 to 1.7; *d* = –0.6), although CIs were wide. Three serious adverse events were deemed unrelated to participation (CBT = 2, TAU = 1).

**Conclusions:**

CBT for nightmares is feasible and may be efficacious for treating nightmares and comorbid insomnia for patients with persecutory delusions. It shows promise on paranoia but potentially not on suicidal ideation.

## Abstract (fr)

**Objectif:**

Les cauchemars sont relativement communs chez les patients souffrant de psychose, mais ils sont rarement évalués ou traités. Les cauchemars peuvent nourrir les délires de persécution en incarnant les peurs par des détails sensoriels abondants. Nous avons testé les bénéfices potentiels de la thérapie cognitivo-comportementale (TCC) axée sur l’imagerie pour les cauchemars relativement à la gravité des cauchemars et aux délires de persécution.

**Méthode:**

Cet essai pilote à l’insu de l’évaluateur et à groupe parallèle a randomisé 24 participants ayant des cauchemars et des délires de persécution pour qu’ils reçoivent une TCC pour cauchemars administrée durant 4 semaines en plus du traitement habituel (TH) ou le TH seulement. Les évaluations ont eu lieu à 0 semaine, 4 semaines (fin du traitement) et au suivi de 8 semaines. Les résultats de faisabilité évaluaient la participation à la thérapie, les techniques utilisées, la satisfaction et l’attrition. Le principal résultat de l’efficacité évaluait la gravité des cauchemars à la 4^e^ semaine. Les analyses portaient sur l’intention de traiter, estimant l’effet du traitement avec des intervalles de confiance (IC) à 95%.

**Résultats:**

Tous les participants ayant eu la TCC complète (moyenne = 4,8 séances, ET = 0,6) ont exprimé une satisfaction élevée, et 20 (83%) participants ont répondu à toutes les évaluations. Comparativement au TH, la TCC entraînait de grandes améliorations des cauchemars (différence moyenne ajustée = 7,0; IC à 95% –12,6 à –1,3, *d* = –1,1) et de l’insomnie (6,3; IC à 95% 2,6 à 10,0, *d* = 1,4) à la 4^e^ semaine. Les gains se maintenaient au suivi. L’idéation suicidaire n’était pas exacerbée par la TCC mais demeurait stable au suivi, comparativement au TH où elle était réduite au suivi (6,8; IC à 95% 0,3 à 3,3, *d* = 0,7). La TCC entraînait des réductions de la paranoïa (–20.8; IC à 95% –43.2 à 1,7, *d* = –0,6), même si les IC étaient larges. Trois effets indésirables sérieux étaient jugés sans relation à la participation (TCC = 2, TH = 1).

**Conclusions:**

La TCC pour les cauchemars est faisable et peut traiter efficacement les cauchemars et l’insomnie comorbide chez les patients souffrant de délire de persécution. Elle est prometteuse pour la paranoïa, mais peut-être pas pour l’idéation suicidaire.

Nightmares depict vivid and highly distressing mental imagery that interrupts restorative sleep. Studies have reported prevalence rates of problematic nightmares ranging between 9% and 55%^1–3^ in patients experiencing psychosis, compared with 2% to 8% of the general population.^4^ Yet nightmares are almost never assessed or treated. Nightmares may both directly and indirectly maintain persecutory beliefs. The direct route is that they portray paranoid fears in rich sensory detail (e.g., the patient experiences being attacked), eliciting a similar neural response as perception of real events.^5,6^ These nightmares are therefore described as seeming real, leaving the patient acutely distressed on waking, which in turn reinforces the fear. In the general population, the content of nightmares most commonly involves imminent physical danger,^4^ and the most common emotion is fear.^7^ This fear might be heightened in a group with persecutory delusions. The indirect route is that nightmares interrupt sleep, triggering negative affect, which is known to exacerbate psychotic experiences.^8^ This study set out to pilot a cognitive behavioural therapy (CBT) treatment for nightmares and assess the effect on paranoia.

Whilst nightmares are associated with a range of negative psychiatric outcomes,^9^ there is a particularly strong association with suicidal ideation. Longitudinal studies in samples without psychosis show that frequent nightmares are associated with 8 times greater odds of later suicidal ideation,^10^ suicide attempts,^11^ and 57% greater risk of death by suicide.^12^ In a sample with schizophrenia, nightmares were not an individual predictor of suicide attempts but, in conjunction with insomnia, led to an 11-fold increase in risk of a suicide attempt.^1^ Whether nightmares are an epiphenomenon or causally related to suicidal ideation is yet to be established. If a causal relationship holds, one would predict a reduction in suicidal ideation either concomitant with a reduction in nightmares or at later follow-up. To our knowledge, only one previous pilot randomized controlled trial (RCT) assessed the effect on suicidal ideation of treating nightmares.^13^ The prazosin treatment unexpectedly increased nightmare severity, and confidence intervals (CIs) for suicidal ideation were in the range of an increasing or decreasing effect. Further research is clearly warranted to elucidate whether nightmares cause or exacerbate suicidal ideation.

The current evaluation was a pilot RCT testing brief CBT for nightmares, compared with treatment as usual (TAU) for patients with persecutory delusions. The primary aims were to 1) assess feasibility and acceptability of the intervention and attrition across the follow-up period and 2) gain initial efficacy data for the impact of the CBT for nightmares intervention on overall nightmare severity (the primary efficacy outcome). Piloting and feasibility assessment were therefore integrated within this 1 trial.^14^ The most established technique for treating nightmares is imagery rescripting (IR).^15–17^ It is recommended in best practice guidelines^15^ and leads to moderate reductions in nightmare frequency compared with controls. IR is a form of imagery-based cognitive therapy in which the patient directly transforms his or her mental images related to distress, for example, by changing the outcome.^18,19^ Based on our learnings from a case series, IR was the core treatment technique,^20^ supplemented with CBT techniques targeting novel potential causal factors for nightmares^21^ (e.g., reducing worry and oversleeping). The key efficacy hypothesis was that CBT for nightmares in addition to TAU would reduce nightmare severity compared with TAU.

## Materials and Methods

### Participants

Twenty-four participants were recruited from Oxford Health National Health Service (NHS) Foundation Trust (*n* = 22) and Central and North West London NHS Foundation Trust (*n* = 2). All were referred by their secondary mental health care coordinator or psychiatrist. The inclusion criteria were 1) experiencing a current chronic problem with distressing nightmares (1 nightmare per week, which was at least moderately distressing, 4 out of 7 on a Likert scale, and experienced for 3 months), 2) a clinical diagnosis of nonaffective psychosis (schizophrenia, schizoaffective disorder, delusional disorder, or psychosis not otherwise specified), 3) reporting a current persecutory delusion meeting the criteria defined by Freeman and Garety^22^ and a score of 33 or above on part A or B of the Green et al.^23^ Paranoid Thoughts Scale, 4) aged 18 to 65 years, and 5) on stable medication (both drug and dose) for at least 4 weeks and no planned medication changes at the point of screening. The exclusion criteria were 1) nightmares that were considered a side effect of medication by the treating psychiatrist; 2) currently receiving CBT or due to commence during the trial period; 3) high risk of sleep apnea indicated by a score of ≥5 on the STOP-BANG questionnaire,^24,25^ with no history of having a full assessment and/or treatment (if the participant was receiving optimal treatment for apnoea, or further NHS assessment resulted in no diagnosis, he or she was invited to take part); 4) a primary diagnosis of personality disorder, alcohol or substance dependency, organic syndrome, or learning disability; or 5) a command of spoken English inadequate for completing questionnaire measures and CBT.

### Design

This was a parallel-group pilot RCT testing brief CBT for nightmares in addition to TAU versus TAU. All participants in the TAU group were offered the full CBT course after their 8-week assessment. Participants were randomized using simple randomization with a ratio of 1:1. The randomization schedule was generated using www.randomisation.com, with randomly varying block sizes. A person independent from the study team generated the allocation sequence and placed each allocation into an opaque sealed envelope prior to recruitment starting.

Two graduate psychologists (SR, KT) took informed consent and completed trial assessments at weeks 0 (baseline), 4 (end of therapy), and 8 (follow-up). A clinical psychologist (BS) informed the participants and their NHS care team of the allocation outcome after completion of the baseline assessment. Research assessors remained blind to allocation (single-blind study). Steps to avoid revealing the allocation status included the assessors not accessing participants’ medical records following randomization, the trial team reminding the participants and their NHS care team not to reveal allocation to the assessor, and the psychologist concealing the diary and whereabouts from the assessors.

Where a blind was broken for the main assessor, a second blind assessor completed that participant’s assessments and rated the time budget measure. This occurred for 1 participant at the week 4 assessment and two further participants at week 8 (all in the CBT group).

The study received NHS ethical approval (15/SC/0502) and was preregistered (ISRCTN12668007). No changes were made to methods after commencement of the trial.

### Interventions

CBT for nightmares was administered by one clinical psychologist (BS) with supervision from a consultant clinical psychologist (DF) and occasional specialist input regarding IR (EH, AG). Therapy sessions lasted around 1 hour and took place over a 4-week window. Workbook-style manuals written by BS, EH, and DF were shared between the participant and therapist to increase adherence to the protocol. These were used flexibly depending on patient preference. The first session began with psychoeducation about nightmares, sharing patient accounts, and key maintenance factors were identified through a nightmare-specific assessment. IR was the key technique and offered to all participants. In this imagery-focussed cognitive therapy approach, participants worked to change the outcome of their nightmare (as if rescripting the end of a film) to create a more benign meaning. For example, if the distressing meaning is that “no one is helping me” to escape my attacker, the patient may change the ending such that he or she experiences someone helping and caring for him or her. This change of ending is planned verbally first, and then critically, details are elicited in sensory modalities (imagination).^26^ A guided imagery recording and written summary were created for each participant to aid imaginary rehearsal between sessions. Subsequent techniques were chosen based on the formulation. These could have included 1) reducing presleep hyperarousal and negative thought content (relaxation, limiting worry and voices), 2) reducing fear of nightmares by increasing coping skills (grounding techniques, writing a compassionate message from the participant’s daytime self), 3) reducing preoccupation related to the nightmare (interrupting thoughts in bed by getting up and winding down in a different room), and 4) stabilizing rapid eye movement sleep across nights (reducing oversleeping or alcohol use and increasing physical activity). Therapy ended with relapse prevention work.

All participants continued with their NHS health care. This typically included antipsychotic medication, regular contact with a care coordinator, and medical reviews. When there were significant concerns regarding suicidal ideation, often elicited through trial assessments, NHS teams referred the participant for more intensive “step-up support,” typically involving daily contact until the acute risk resolved.

### Feasibility and Acceptability Outcomes

A therapy log recorded CBT techniques delivered and number of sessions used. An independent graduate psychologist (neither the assessor nor the therapist) asked each participant in the treatment group, “Overall, how satisfied were you with the therapy you received?” Responses were recorded using a visual analog scale from 0 (not at all satisfied) to 10 (very satisfied). The number who completed the follow-up assessments was recorded, with reasons for dropout where available.

### Primary Efficacy Outcome Measure

The Disturbing Dream and Nightmare Severity Index (DDNSI)^27^ is a 5-item self-report scale assessing nightmare severity. Questions relate to nights per week with nightmares, nightmare frequency, awakenings, severity of nightmare problem, and the intensity of nightmares. The total score ranges from 0 to 37, with higher scores indicating a more severe problem. The internal consistency of the scale in a large sample of over 3000 students was very good (α = 0.91).^28^

### Secondary Efficacy Outcomes

Other sleep outcome measures included the Sleep Condition Indicator (SCI),^29^ an 8-item self-report measure of insomnia (total score range 0-32). Higher scores indicate better sleep. The Pittsburgh Sleep Quality Index (PSQI)^30^ assessed self-reported sleep quality. The total score ranges from 0 to 21 (worse sleep).

Affective symptoms were measured by the Depression, Anxiety and Stress Scale, 21-item version (DASS-21).^31^ Twenty-one items are rated from 0 to 4 and are totalled to create 3 subscales, ranging from 0 to 21 (high).

The Green et al.^23^ Paranoid Thoughts Scale (GPTS) assessed paranoia via 32 self-report items. Response options range from 1 (not at all) to 5 (totally). The total score ranges from 32 to 160 (high). The Cardiff Anomalous Perceptions Scale (CAPS)^32^ assessed 32 anomalous experiences via self-report. The total number of experiences endorsed (0-32) is reported.

Other psychiatric symptoms were assessed using the Beck Suicide Scale (BSS),^33^ a 21-item self-report measure of suicidal ideation. Higher scores indicate increased suicidal ideation. The Brief Dissociative Experiences Scale (DES-B)^34^ assessed dissociative experiences. An average score (ranging from 0 to 4 = extreme) is calculated by dividing the total score by 8 items.

Broader well-being measures included the Warwick-Edinburgh Mental Wellbeing Scale (WEMWBS).^35^ Fourteen self-report items are rated from 1 to 5. These are totalled to create a score ranging from 14 to 70 (high). The Time Budget Questionnaire^36^ assessed activity levels, with scores ranging from 0 to 112 (high activity).

Alternative measures of nightmares included the Oxford Nightmare Severity Scale. This is a new measure of nightmare severity assessed over a 2-week time frame. Three filter questions assess nightmare frequency. If a participant endorses experiencing at least one nightmare, 3 subscales are completed. Each subscale includes 15 items that assess 1) nightmare-related distress, 2) preoccupation, and 3) impairment. Items are rated from 0 (not at all) to 4 (very much). Subscale scores range from 0 to 60. All 45 items are summed to create a total severity score (range, 0-180). Higher scores indicate greater severity.

A prospective nightmare log was kept by participants, completed each morning using pen and paper over a 7-day period. They assessed 1) the number of nightmares experienced, 2) a distress rating for each (1 = not at all distressing, 10 = extremely distressing), 3) the number of awakenings due to nightmares, and 4) sleep quality (0 = very poor, 4 = very good).

### Medication Use

The defined daily dose (DDD)^37^ was used to convert antipsychotic, mood stabilizer, and anxiolytic medications into an equivalent dose for each participant. The DDD reflects the recommended maintenance dose (long-term therapeutic dose) of a medication and is the gold standard measure for comparing drug utilization. The number of pro re nata (PRN, “taken when necessary”) medications prescribed was also measured.

### Adverse Events

Serious adverse events (SAEs) were defined as 1) deaths, 2) suicide attempts, 3) serious violent incidents, 4) admissions to secure units, and 5) formal complaints about the therapy. SAEs were recorded throughout the duration of the trial, and upon completion of the trial, medical records of all participants were systematically reviewed.

### Statistical Analyses

A detailed statistical analysis plan was completed prior to conducting the analysis. A sample size of 24 was chosen to meet the primary objective of assessing feasibility and acceptability, rather than statistically significant between-group changes. A sample of 24 allowed a 95% CI of the proportion of participants who complete follow-up to have width of 35%, if 80% complete the follow-up.

The histograms for the residuals of all efficacy outcome measures were visually assessed and deemed sufficiently normal for subsequent analysis. Adjusted treatment difference and 95% CIs were estimated using a linear mixed-effects model, which accounts for repeated measures over time. The baseline score of each variable was added as a covariate in the model. Assessment point (weeks 4 and 8), outcome of randomization, and an interaction between assessment point and randomized group were included as fixed effects to allow estimation of the treatment effect at the 2 time points. Random intercepts were included to account for repeated measurements on participants. Given the objectives of this pilot RCT, the analysis plan did not include reporting of *p* values. Instead, the treatment effect provides initial efficacy data in preparation for a larger trial. If the 95% CI spans 0, we can be less sure of the direction of the true treatment difference (or if there is an effect at all).

Standardized effect sizes are reported using Cohen’s *d* (adjusted mean difference between groups/pooled baseline standard deviation). Analysis began after the final assessment was complete, following intention-to-treat principles. It was conducted by BS using SPSS for Windows (version 25)^38^ and validated by a trial statistician (AN).

## Results

**Figure 1.**
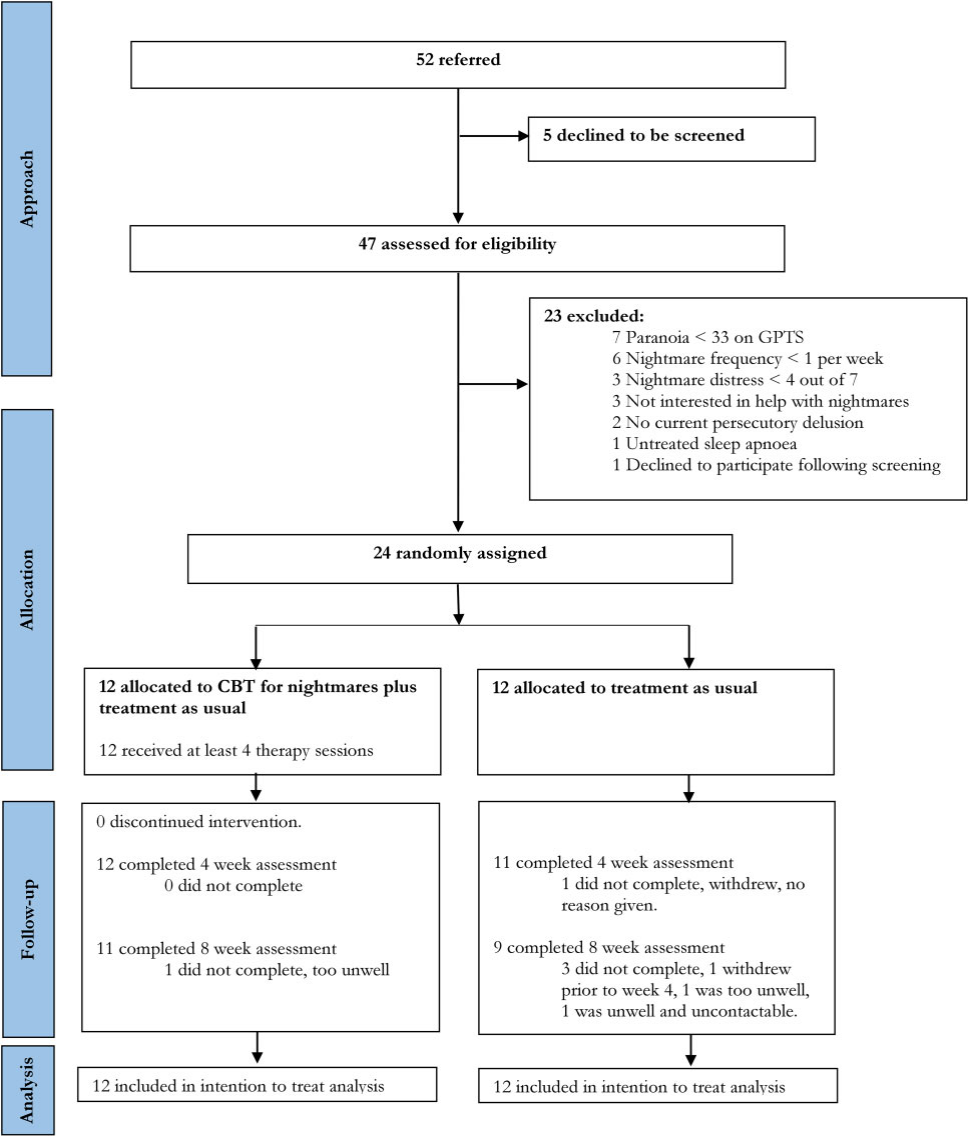
CONSORT flow diagram. CBT, cognitive behavioural therapy; TAU, treatment as usual.

Recruitment took place between February 2016 and March 2018, with breaks for staff leave and commitment to other trials. Twenty-four participants were randomly allocated to CBT for nightmares in addition to TAU (*n* = 12) or TAU (*n* = 12). Participant flow is described in the CONSORT diagram (Figure 1).

### Baseline Demographic and Clinical Characteristics

The 2 groups were broadly balanced with respect to the primary efficacy outcome (DDNSI), demographics, and clinical characteristics (Table 1). Most participants were white British, single, and unemployed; had a diagnosis of schizophrenia or schizoaffective disorder; and were supported by adult mental health teams. All but one participant (in the CBT group) fell below the SCI cutoff and therefore had insomnia disorder. Three-quarters of participants reported that they had attempted suicide at least once in their lifetime (prior to participation in the study).

**Table 1.** Baseline Demographics and Clinical Characteristics (*N* = 24).

CBT for Nightmares (*n* = 12)	Treatment as Usual (*n* = 12)	
Age, mean (SD), y	43 (12)	39 (13)
Sex		
Female	5 (42)	5 (42)
Male	7 (58)	7 (58)
Ethnicity and citizenship		
White British	10 (83)	9 (75)
Mixed/multiple ethnic groups—White and Black African	1 (8)	0 (0)
Black Caribbean	1 (8)	0 (0)
Other	0 (0)	1 (8)
Asian/Asian British—Indian	0 (0)	1 (8)
Asian/Asian British—Pakistani	0 (0)	1 (8)
Marital status		
Single	10 (83)	7 (58)
Married	2 (17)	3 (25)
Cohabiting	0 (0)	1 (8)
Divorced/separated	0 (0)	1 (8)
Employment status		
Unemployed	9 (75)	10 (83)
Self-employed	0 (0)	1 (8)
Student	2 (17)	1 (8)
Retired	1 (8)	0 (0)
Diagnosis		
Schizophrenia	6 (50)	3 (25)
Schizoaffective disorder	5 (42)	4 (33)
Delusional disorder	0 (0)	1 (8)
Psychosis not otherwise specified	1 (8)	4 (33)
Clinical team		
Adult mental health team	10 (83)	9 (75)
Adult inpatient ward	1 (8)	0 (0)
Early intervention service	1 (8)	3 (25)
Medication use, mean (SD)		
Antipsychotic DDD	1.5 (0.6)	1.5 (1.0)
Mood stabilizer DDD	0.1 (0.2)	0.1 (0.3)
Anxiolytic DDD	0.0 (0.1)	0.1 (0.3)
Antidepressant DDD	1.0 (1.3)	2.5 (1.7)
Nightmare severity (DDNSI), mean (SD)	21.6 (6.9)	23.0 (6.4)
Number of nightmares per week (median, IQR)	4.0 (2.8-7.5)	3.5 (3.0-8.0)
Insomnia disorder (SCI cutoff)	11 (92)	12 (100)
Current suicidal ideation (BSS >0)	6 (50)	7 (58)
Number of previous suicide attempts		
Never previously attempted suicide	4 (33)	2 (17)
Attempted suicide once	2 (17)	3 (25)
Attempted suicide 2 or more times	6 (50)	7 (58)

Data are *n* (%) unless otherwise specified.

BSS, Beck Suicide Scale; CBT, cognitive behavioural therapy; DDD, defined daily dose of medication; DDNSI, Disturbing Dream and Nightmare Severity Index; IQR, interquartile range; SCI, sleep condition indicator.

### Medication Use

Baseline medication use is shown in Table 1. The groups were broadly balanced with respect to antipsychotics, mood stabilizers, and anxiolytic medication. Whilst the DDD of antidepressant medication was at the long-term therapeutic dose in the CBT for nightmares group, it was 2.5 times that dose in the TAU group. There were no changes in the mean DDD of any medication from baseline to 4 or 8 weeks. Prescriptions of PRN medication were very low across the groups (Suppl. Table S1).

### Feasibility and Acceptability Outcomes

#### Sessions used

All 12 participants who were offered CBT for nightmares completed the course (at least 4 sessions ending in relapse prevention) within the 4-week therapy window. Four participants received 4 sessions, 7 completed 5 sessions, and 1 participant received 6 sessions (mean [SD], 4.8 [0.6]).

#### Key techniques delivered

The CBT techniques used in the treatment group are shown in Table 2.

**Table 2.** Cognitive Behavioural Therapy Techniques Used within the Treatment Group.

Technique	Number of Participants (*n* = 12)	%
Psychoeducation	12	100.0
Relapse prevention	12	100.0
Imagery rescripting	11	91.7
Reducing presleep hyperarousal: relaxation activities	8	66.7
Reducing fear of nightmares: compassionate message	8	66.7
Stabilizing REM sleep: limiting sleep duration	6	50.0
Reducing presleep hyperarousal: limiting presleep worry	5	41.7
Interrupting nightmare-related preoccupation: 15-minute rule	5	41.7
Stabilizing REM sleep: increasing positive activity	3	25.0
Reducing fear of nightmares: grounding techniques	3	25.0

REM, rapid eye movement.

#### Therapy satisfaction

Eleven out of 12 participants provided a therapy satisfaction rating to an independent assessor. The median score was 9 out of 10 (interquartile range [IQR], 6.75-10).

### Primary Efficacy Outcome

The TAU group remained stable in their nightmare severity across time. Compared with TAU, the CBT for nightmares had a treatment benefit on nightmare severity in the large effect size range at weeks 4 and 8 (Table 3). The 95% CIs for the adjusted treatment effects do not cross 0.

**Table 3.** Scores for Primary and Secondary Efficacy Outcome Measures.

	CBT for Nightmares (*n* = 12), Mean (SD)	*n*	Treatment as Usual (*n* = 12), Mean (SD)	*n*	Adjusted Mean Difference (95% CI)	Effect Size (*d*)
Primary outcome measure						
Nightmare severity (DDNSI)						
Week 0	21.6 (6.9)	12	23.0 (6.4)	12		
Week 4	14.2 (8.8)	12	22.6 (7.1)	11	–7.0 (–12.6 to –1.3)	–1.06
Week 8	12.6 (8.6)	11	22.1 (8.2)	9	–6.7 (–12.4 to –0.9)	–1.02
Secondary outcome measures						
Insomnia (SCI-8 item)						
Week 0	8.6 (4.5)	12	7.6 (4.6)	12		
Week 4	15.2 (7.7)	12	7.4 (5.0)	10	6.3 (2.6 to 10.0)	1.40
Week 8	14.9 (6.9)	11	8.4 (6.8)	9	4.3 (0.4 to 8.1)	0.95
Sleep quality (PSQI)						
Week 0	12.2 (4.4)	12	12.7 (3.5)	12		
Week 4	10.2 (3.8)	12	12.5 (4.3)	10	–1.9 (–4.1 to 0.4)	–0.48
Week 8	9.6 (3.9)	11	11.8 (3.8)	9	–1.7 (–4.0 to 0.6)	–0.43
Depression (DASS-21)						
Week 0	13.2 (5.5)	12	14.3 (5.7)	12		
Week 4	10.8 (7.0)	12	13.8 (6.5)	10	–0.9 (–4.5 to 2.8)	–0.15
Week 8	11.5 (5.8)	11	10.6 (5.3)	9	3.3 (–0.5 to 7.1)	0.60
Anxiety (DASS-21)						
Week 0	10.8 (5.1)	12	14.5 (5.2)	12		
Week 4	7.4 (5.8)	12	13.3 (5.4)	10	–2.4 (–5.0 to 0.2)	–0.45
Week 8	7.6 (5.3)	11	11.0 (4.6)	9	0.4 (–2.3 to 3.1)	0.08
Stress (DASS-21)						
Week 0	11.8 (4.0)	12	15.5 (3.7)	12		
Week 4	9.0 (5.6)	12	15.9 (3.1)	10	–2.6 (–6.1 to 0.9)	–0.61
Week 8	10.4 (6.2)	11	13.3 (5.1)	9	1.1 (–2.5 to 4.7)	0.27
Paranoia (GPTS)						
Week 0	101.2 (35.7)	12	109.8 (33.9)	12		
Week 4	75.3 (37.0)	12	109.0 (32.3)	10	–20.8 (–43.2 to 1.7)	–0.60
Week 8	68.5 (39.4)	11	100.7 (35.5)	9	–18.5 (–41.0 to 4.0)	–0.54
Hallucinations—total endorsement (CAPS total)						
Week 0	17.7 (7.7)	12	18.8 (7.1)	12		
Week 4	15.5 (7.7)	12	16.8 (7.3)	10	0.9 (–4.0 to 5.8)	0.12
Week 8	15.8 (7.8)	11	16.7 (10.1)	9	0.8 (–4.2 to 5.8)	0.11
Suicidal ideation (BSS)						
Week 0	6.5 (8.4)	12	10.5 (10.6)	12		
Week 4	5.7 (7.6)	12	5.9 (7.5)	10	2.8 (–3.5 to 9.2)	0.30
Week 8	7.2 (10.9)	11	2.7 (5.7)	9	6.8 (0.3 to 13.3)	0.71
Dissociation—average (DES-B average)						
Week 0	1.7 (0.7)	12	2.1 (0.8)	12		
Week 4	1.5 (0.9)	12	2.4 (0.6)	10	–0.7 (–1.4 to 0.1)	–0.84
Week 8	1.2 (1.0)	11	1.9 (0.8)	9	–0.4 (–1.2 to 0.3)	–0.54
Emotional well-being (WEMWBS)						
Week 0	38.6 (7.5)	12	34.0 (9.7)	12		
Week 4	44.5 (12.2)	12	34.2 (10.4)	10	3.8 (–4.6 to 12.2)	0.43
Week 8	44.1 (12.4)	11	41.0 (9.9)	9	–4.2 (–12.9 to 4.6)	–0.47
Activity levels (time budget)						
Week 0	61.9 (14.6)	12	60.1 (10.0)	11		
Week 4	65.7 (18.0)	11	62.1 (9.6)	10	1.1 (–7.7 to 9.9)	0.09
Week 8	63.1 (16.1)	11	62.6 (7.8)	9	–1.7 (–10.7 to 7.3)	0.14

All analyses controlled for baseline score for that variable.

BSS, Beck Suicide Scale; CAPS, Cardiff Anomalous Perceptions Scale (higher scores indicate more severe symptoms); CBT, cognitive behavioural therapy; CI, confidence interval; DASS-21, Depression Anxiety and Stress Scale–21-item version (higher scores indicate more severe symptoms); DDNSI, Disturbing Dream and Nightmare Severity Index (higher scores indicate a more severe problem with nightmares); DES-B, Brief Dissociative Experiences Scale; GPTS, Green Paranoid Thoughts Scale (higher scores indicate more severe symptoms); PSQI, Pittsburgh Sleep Quality Index (lower scores indicate better sleep quality); SCI-8, Sleep Condition Indicator–8-item version (higher scores indicate less insomnia); WEMWBS, Warwick-Edinburgh Mental Wellbeing Scale.

### Secondary Efficacy Outcome

The CBT for nightmares group had a treatment benefit on insomnia in the large effect size range at weeks 4 and 8. The CIs do not overlap 0 (Table 3). For paranoia, sleep quality, affective symptoms, dissociation, emotional well-being, and additional nightmare measures by week 4 (end of therapy), the effects were in the direction of CBT for nightmares improving outcomes. For paranoia, sleep quality, dissociation, and additional nightmare measures, the direction of this effect remained at follow-up (Tables 3 and 4).

**Table 4.** Scores for Additional Nightmare Outcome Measures.

	CBT for Nightmares, Mean (SD)	*n*	Treatment as Usual, Mean (SD)	*n*	Adjusted Mean Difference (95% CI)	Effect Size (*d*)
Nightmare severity (ONSS total)						
Week 0	108.5 (26.8)	12	135.2 (23.8)	12		
Week 4	70.9 (42.1)	12	121.0 (29.4)	10	–19.3 (–50.3 to 11.7)	–0.8
Week 8	56.6 (45.0)	11	113.7 (38.8)	9	–22.4 (–54.0 to 9.2)	–0.9
Nightmare-related distress (ONSS distress)						
Week 0	37.3 (8.3)	12	45.1 (8.5)	12		
Week 4	25.0 (15.1)	12	41.1 (9.9)	10	–8.2 (–20.6 to 4.2)	–1.0
Week 8	20.7 (17.0)	11	37.2 (13.5)	9	–7.7 (–20.4 to 4.9)	–0.9
Preoccupation with nightmares (ONSS preoccupation)						
Week 0	40.0 (7.8)	12	47.4 (6.5)	12		
Week 4	25.2 (14.6)	12	42.5 (9.0)	10	–11.3 (–22.8 to 0.2)	–1.6
Week 8	17.8 (13.3)	11	41.3 (12.4)	9	–16.5 (–28.3 to –4.7)	–2.3
Impairment related to nightmares (ONSS impairment)						
Week 0	31.3 (13.0)	12	42.7 (11.9)	12		
Week 4	20.6 (14.7)	12	37.4 (13.0)	10	–4.7 (–14.5 to 5.0)	–0.4
Week 8	18.0 (16.2)	11	35.1 (15.4)	9	–3.4 (–13.4 to 6.6)	–0.3

All analyses controlled for baseline score for that variable.

CBT, cognitive behavioural therapy; CI, confidence interval; ONSS, Oxford Nightmare Severity Scale.

For hallucinations and activity levels, there was no effect favouring either group. The TAU group had a much higher starting mean suicidal ideation at baseline that decreased, whereas the suicidal ideation for the CBT group remained relatively stable from week 0 to week 8. There was a small effect size improvement in suicidal ideation for the TAU group at week 4, but the CIs for the treatment effects are wide and include zero. By week 8, recovery in suicidal ideation for the TAU group resulted in a medium effect size improvement when compared to the CBT group. CIs do not cross zero. Post hoc exploratory analysis revealed that of the 11 participants who reported no suicidal ideation at baseline (6 participants = CBT group, 5 participants = TAU group), one participant reported new onset of suicidal ideation within the trial period. This was a TAU group participant at week 8 (supplementary materials).

At week 4, the effect sizes for affective symptoms and well-being were in the direction of CBT for nightmares improving outcomes, albeit with CIs overlapping 0. By week 8, both groups had improved on measures of depression and stress, but the direction of effect favoured more recovery in the TAU group. The 95% CIs, however, overlap 0. There was no effect for anxiety favouring either group at week 8.

### Serious Adverse Events

There were 3 SAEs, all of which were suicide attempts (2 = CBT for nightmares group, 1 = TAU group). An adverse event report was written, and each was assessed by the chief investigator as unrelated to participation in the trial based on 1) the temporal relationship between trial procedures and adverse events, 2) participant report of reasons for suicidal ideation, and 3) reasons for suicidal ideation obtained from medical notes. Two participants continued participation in the trial, and 1 chose to not complete the final assessment owing to being unwell.

## Discussion

This was the first RCT to assess a brief CBT intervention targeting nightmares specifically for patients with psychosis. Methodological rigour was high for a pilot trial: random allocation was used, assessments were successfully blinded, all participants completed therapy, follow-up rates were high, and therapy satisfaction was collected by an independent assessor.

A large reduction in nightmare severity was found in the CBT group compared with TAU. This effect size is similar to or indeed slightly larger than other trials of IR tested in groups without psychosis.^39^ This questions the common recommendation that comorbid psychosis may be a contraindication for IR. A large reduction in insomnia was also found following CBT compared with TAU. This may be due to a reduction in nightmare-related awakenings, reduced fear of sleep, or sleep window stabilization. The fact that this 4-week intervention affects insomnia as well as nightmares is highly promising given that the 2 conditions are highly comorbid in the psychosis population, although this of course requires replication in an adequately powered trial.^2^

The trial also collected data on a new outcome in the evaluation of the effects of the treatment of nightmares: persecutory delusions. The intervention that focused on the content of nightmares via IR, without any behavioural tests of persecutory beliefs, led to moderate reductions in paranoia at weeks 4 and 8 compared with TAU. This is consistent with the view that nightmares may maintain the persecutory fear, although further research in an adequately powered trial is required to establish effect sizes more precisely. Focusing on “dreams” rather than the persecutory belief per se allowed an opportunity to be creative, encouraging belief flexibility by considering alternative safe endings. Bringing the perceptual system online using guided imagery allowed participants to try out this new safe ending and experience the associated sensory and emotional detail.^40,41^ The approach was well received by participants, as indicated by 100% therapy completion and high therapy satisfaction.

An unexpected finding was the recovery of suicidal ideation for the TAU group compared with stable but not exacerbated suicidal ideation in the CBT group. The CBT group also showed descriptively slower recovery in depression at week 8 compared to TAU. The sample size is clearly small, particularly so for suicidal ideation given that only half of the participants scored above 0 at baseline, so this result should be interpreted cautiously. However, possible interpretations include the following: 1) incomplete remission in the nightmares for some participants in the CBT group may prolong hopelessness and hence maintain suicidal ideation; 2) focusing on nightmare imagery in CBT may heighten awareness of suicidal “fast-forward” imagery, which was not the focus of the intervention but is a risk factor for later ideation^42,43^; or 3) the TAU group received the CBT intervention immediately after their 8-week assessment and hence may have been more optimistic at that point. Given that two-thirds of the sample had previously attempted suicide and around half reported current ideation, it is clearly an important clinical issue. Future studies should carefully monitor it and any associated imagery.

There are clear limitations to the current trial. The predetermined sample size was not designed to detect significant differences; hence, future adequately powered studies are required to establish the efficacy of the intervention. The TAU group had a higher dose of antidepressant medication throughout the trial, and antidepressants have been linked with alterations in rapid eye movement sleep. As is typical of pilot trials, recruitment took place at one university centre with one therapist, which limits generalizability. No assessment was made of therapist adherence to CBT. The measure of hallucinations (CAPS) included a range of hallucinatory experiences (e.g., auditory and visual domains). A clinical observation was that in some cases, nightmares played out threats from voices, but the CAPS was likely not sensitive enough to abusive voices specifically to detect any potential change. There was no active control group, and hence it is not possible to attribute the changes in nightmares to the CBT specifically over and above nonspecific effects of therapy. Whilst this is an appropriate design for a trial of this size,^44^ later efficacy trials would benefit from comparison with an active control (e.g., befriending).

The current results suggest that a brief, targeted CBT intervention for nightmares using IR is feasible and may lead to substantial improvements in nightmares and insomnia. This therapy could be a standalone intervention or form part of a longer piece of work targeting maintenance factors for persecutory delusions. A larger trial is warranted to establish efficacy data, but suicidal ideation requires careful monitoring.

## Supplemental Material

Supplemental Material, 847422_Supplementary_materials - Cognitive Behavioural Therapy for Nightmares for Patients with Persecutory Delusions (Nites): An Assessor-Blind, Pilot Randomized Controlled Trial Supplemental Material, 847422_Supplementary_materials for Cognitive Behavioural Therapy for Nightmares for Patients with Persecutory Delusions (Nites): An Assessor-Blind, Pilot Randomized Controlled Trial by Bryony Sheaves, Emily A. Holmes, Stephanie Rek, Kathryn M. Taylor, Alecia Nickless, Felicity Waite, Anne Germain, Colin A. Espie, Paul J. Harrison, Russell Foster and Daniel Freeman in The Canadian Journal of Psychiatry
