# Supplementary material for: Cognitive Behavioural Therapy for Nightmares for Patients with Persecutory Delusions (Nites): An Assessor-Blind, Pilot Randomized Controlled Trial
Source: Can J Psychiatry. 2019 May 26;64(10):686–96. doi: 10.1177/0706743719847422 (PMC6783669; doi:10.1177/0706743719847422)
Supplement: Supplemental Material, 847422_Supplementary_materials - Cognitive Behavioural Therapy for Nightmares for Patients with Persecutory Delusions (Nites): An Assessor-Blind, Pilot Randomized Controlled Trial [file 847422_Supplementary_materials.pdf]

Appendices

SUPPPLEMENTARY TABLE A1. PRO RE NATA (PRN) MEDICATION USE OVER TIME

	CBT for nightmares (n=12)	Treatment as usual (n=12)
PRN antipsychotics		
Week 0	1 (8%)	0
Week 4	1 (8%)	1 (8%)
Week 8	1 (8%)	1 (8%)
PRN mood stabilisers		
Week 0	0 (0%)	0 (0%)
Week 4	0 (0%)	0 (0%)
Week 8	0 (0%)	0 (0%)
PRN anxiolytics		
Week 0	2 (17%)	0 (0%)
Week 4	2 (17%)	1 (8%)
Week 8	2 (17%)	2 (17%)
PRN anti-depressants		
Week 0	0 (0%)	0
Week 4	0 (0%)	1 (8%)
Week 8	0 (0%)	1 (8%)
PRN hypnotics		
Week 0	0 (0%)	0 (0%)
Week 4	0 (0%)	0 (0%)
Week 8	0 (0%)	1 (8%)

Data are n (%).

SUPPLEMENTARY TABLE A2. NIGHTMARE LOG SCORES OVER TIME

	CBT for nightmares	n	Treatment as usual	n	Adjusted mean difference (95% CI)	Effect size (d)
Nightmare log total frequency						
Week 0	7.4 (5.8)	11	9.9 (7.9)	12		
Week 4	4.4 (4.8)	9	8.9 (10.7)	8	-2.6 (-5.8; 0.6)	-0.4
Week 8	3.7 (3.4)	10	12.5 (9.4)	6	-4.8 (-8.3; -1.3)	-0.7
Nightmare log average distress						
Week 0	6.6 (2.4)	11	6.4 (1.8)	12		
Week 4	3.9 (2.9)	9	6.3 (1.7)	9	-2.2 (-4.5; 0.3)	-1.0
Week 8	4.4 (2.9)	10	5.9 (1.5)	6	-1.6 (-4.1; 0.9)	-0.8
Nightmare log – total number of awakenings						
Week 0	6.6 (6.1)	11	7.4 (6.6)	12		
Week 4	3.6 (4.9)	8	7.8 (8.6)	9	-3.7 (-7.4; 0.1)	-0.6
Week 8	1.9 (1.7)	9	9.5 (6.7)	6	-3.8 (-8.0; 0.3)	-0.6
Nightmare log – average sleep quality						
Week 0	1.7 (0.9)	11	1.7 (0.7)	12		
Week 4	2.2 (0.9)	9	1.8 (1.1)	9	0.4 (-0.4; 1.2)	0.5
Week 8	2.1 (0.9)	10	1.4 (0.6)	6	0.6 (-0.3; 1.4)	0.7

Data are mean (SD). CI=confidence interval.

SUPPLEMENTARY TABLE A3. DICHOTOMOUS ASSESSMENT OF SUICIDAL IDEATION ACROSS THE TRIAL PERIOD

	Suicidal ideation outcome at week 4 (n=22)				Suicidal ideation outcome at week 8 (n=20)			
	No suicidal ideation		Suicidal ideation		No suicidal ideation		Suicidal ideation	
	CBT for nightmares	Treatment as usual	CBT for nightmares	Treatment as usual	CBT for nightmares	Treatment as usual	CBT for nightmares	Treatment as usual
No suicidal ideation at baseline	6 (27%)	3 (14%)	0 (0%)	0 (0%)	6 (30%)	2 (10%)	0 (0%)	1 (5%)
Suicidal ideation at baseline	0 (0%)	2 (9%)	6 (27%)	5 (23%)	0 (0%)	4 (20%)	5 (25%)	2 (10%)

Data are counts (%). No suicidal ideation indicated by BSS score of 0. Suicidal ideation indicated by BSS >0.
